# The Key Role of Uric Acid in Oxidative Stress, Inflammation, Fibrosis, Apoptosis, and Immunity in the Pathogenesis of Atrial Fibrillation

**DOI:** 10.3389/fcvm.2021.641136

**Published:** 2021-02-26

**Authors:** Yawen Deng, Fei Liu, Xiaolei Yang, Yunlong Xia

**Affiliations:** Department of Cardiology, First Affiliated Hospital of Dalian Medical University, Dalian, China

**Keywords:** uric acid, atrial fibrillation, mechanisms, oxidative stress, inflammation

## Abstract

Atrial fibrillation (AF) is a highly prevalent cardiac arrhythmia that leads to numerous adverse outcomes including stroke, heart failure, and death. Hyperuricemia is an important risk factor that contributes to atrium injury and AF, but the underlying molecular mechanism remains to be elucidated. In this review, we discussed the scientific evidence for clarifying the role of hyperuricemia in the pathogenesis of AF. Experimental and Clinical evidence endorse hyperuricemia as an independent risk factor for the incidence of AF. Various *in vivo* and *in vitro* investigations showed that hyperuricemia might play a critical role in the pathogenesis of AF at different UA concentrations through the activation of oxidative stress, inflammation, fibrosis, apoptosis, and immunity.

## Introduction

Atrial fibrillation (AF) is considered to be the most frequent cardiac arrhythmia and its prevalence is increasing substantially. In 2016, 46.3 million individuals had prevalent AF/atrial flutter globally, and the prevalence of AF has been estimated between 2 and 4% in adults (1). Also, the prevalence of AF is expected to rise more than double in the next three decades, largely owing to the extended life expectancy of the general population, intensifying search for undiagnosed AF (2), and longer survival with chronic conditions (3). AF is associated with a 5-fold risk for stroke and is estimated to cause 15% of all strokes (4), and among various cardiac arrhythmia, AF is receiving significant attention for its contribution to cardiac mortality and morbidity (5).

Chronic diseases such as rheumatic heart disease, hypertension, hyperthyroidism, chronic kidney disease, and diabetes mellitus have all been regarded as risk factors for AF (6, 7). Although the pathophysiology underlying AF remains to be fully elucidated, inflammation and oxidative stress are partially known for their involvement in the pathogenesis of AF (8). Recently, increased focus has been given to the possible mechanism by which hyperuricemia causes AF. Similarly, the link between hyperuricemia and other conditions such as hypertension, metabolic syndrome, diabetes mellitus, and chronic kidney disease has been reported (9–11). Uric acid [UA; 7,9-hihydro-1H-purine-2,6,8(3H)-trione; C5H4N4O3; molecular weight of 168.11 Da] is a heterocyclic organic compound and an end product of purine metabolism in humans. UA acts as an antioxidant and pro-oxidant at its normal and high concentration, respectively (12). Difficulties in determining whether UA acts as a risk marker or a risk factor for AF remained debatable due to the frequent association and intricate relationship with other cardiovascular risk factors. Despite such controversy, the interest in UA has recently resurrected.

A recent review on the role of UA in cardiovascular diseases (CVD) has focused on summarizing the association between uric acid and various cardiovascular diseases, mainly from experimental evidence (13). Also, the review was shallow in the area of AF despite its well-organized content on the relationship between UA and CVD. Bearing in mind the significance of bridging the experimental findings with clinical evidence, it is undoubtedly important to summarize the association of UA with AF based on both experimental and clinical evidence. Therefore, this review will discuss the potential mechanism on how UA involves in AF pathophysiology. In particular, our review will summarize the effects of fibrosis, apoptosis, and immunity on the progress of AF and how elevated UA associated with hypertension and metabolic syndrome aggravates the risk of AF.

## The Experimental Evidence in AF

**Figure 1:**
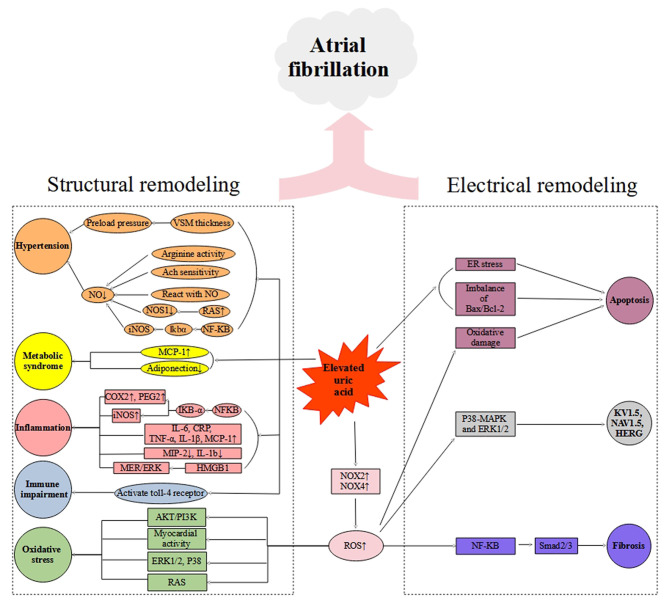
Schematic diagram of putative mechanisms of hyperuricemia-mediated atrial fibrillation. VSM, vascular smooth muscle; Ach, Acetylcholine; NO, Nitric Oxide; RAS, Renin-angiotensin system; NOS1, Nitric Oxide Synthase 1; iNOS, Inducible Nitric Oxide Synthase; MCP-1, Monocyte chemoattractant protein-1; CRP, C-reactive protein; COX-2, Cyclooxygenase-2; PGE2, Prostaglandin 2; IL-6, Interleukin-6; TNF-α, Tumor necrosis factor-α; IL-1β, Interleukin-1β; MIP-2, Macrophage inflammatory protein-2; IL-1b, Interleukin-1b; HMGB1, High Mobility Group Box 1; NOX2, NADPH oxidase 2; NOX4, NADPH oxidase 4.

Bearing in mind that the extensive overlap exists between comorbidities and risk factors of hyperuricemia and arrhythmias, multivariable analyses of epidemiologically collected data cannot substitute proof generated from basic and clinical studies. As such, there is a need for further basic research to establish a causal relationship between UA and AF and to identify the mechanism by which UA is involved in AF pathology. Elevated UA can cause arrhythmia either directly or indirectly, depending on the availability of other risk factors. In this review, we have summarized *in vitro* and *in vivo* studies regarding UA acting on several cellular signaling pathways in Table 1. The summary of the elevated UA induced cardiac remolding related to electrophysiological and structural alterations via various mechanisms, including oxidative stress, inflammation, fibrosis, apoptosis, and immunity is described in Figure 1.

**Table 1:** *In vitro* and *in vivo* studies regarding uric acid acting on several cellular signaling pathways in different hyperuricemia models.

**The types the cells**	**Concentration**	**Acting duration**	**Pathway**	**Animal model**	**References**
Human proximal tubular cell	4,8,16 mg/dl	24, 48 and 72 h	MAPK pathway	No	(14)
Cardiomyocyte	0–15 mg/dl	12, 24, 48 and 72 h	ERK/P38	Yes^a^	(15)
Renal proximal tubule cell	500 μM	8 h	PKC, MAPK, cPLA2, and NF-kB	No	(15)
Human umbilical vein endothelial cells	10, 50, and 100 g/ml	60 min	MEK/Erk pathway	No	(16)
β-cell	5 mg/dL	24 h	NF-kB-iNOS-NO signaling axis.	Yes^b^	(17)
Mouse atrial myocytes	7 mg/dl	24 h	ERK pathway	No	(18)
Pre-adipocyte 3T3-L1 cells	15 mg/dl	5–30 min	p38 and ERK1/2 MAP kinases pathway	No	(19)
Cardiomyocytes	0, 5, 10, and 15 mg/dl	30 min, 1, 8, 16 and 24 h	IRS-PI3K-Akt signaling	No	(20)
Rat cardiac fibroblasts	3-300 μM	24 h	ERK	No	(21)
Interstitial macrophages	3% UA	7 weeks to feed	RAS-NOS1	Yes^c^	(22)
Human vascular smooth muscle cells	6 to 12 mg/dl	1 to 48 h	p38	No	(23)
Vascular smooth muscle cells	2.5 to 10 mg/dL	72 h	MAPK signaling molecules ERK p44/42 and p38 NF-κB	Yes^d^	(24)
Human mesangial cells	8-50 mg/dl	24 h	COX-2 expression and PGE2 synthesis	No	(25)
Pulmonary artery endothelial cells.	2.5–15 mg/dl	24 h	L-arginine-eNOS pathway	No	(26)

a *Intraperitoneal injection of potassium oxonate (300 mg/kg) and intragastric administration of hypoxanthine (500 mg/kg) for 1–2 h to create acute hyperuricemia*.

b *The mouse hyperuricemia model was generated by daily intraperitoneal injection of uric acid (250 mg/kg, Sigma) for 4 weeks*.

c *Mild hyperuricemia was induced in rats by providing a uricase inhibitor- oxonic acid (OA) and marked hyperuricemia were fed with 2% OA and 3% UA in the diet*.

d *OA feeding for rats*.

### UA Participates in the Progress of Hypertension-Induced AF

Clinically, hypertension is an independent risk factor for AF. In the past, it was well-established that UA and nitrate concentrations were positively associated with elevated blood pressure (27). Some researchers also demonstrated that UA, the most abundant antioxidant in plasma, reacts directly with nitric oxide (NO) in a rapidly irreversible reaction resulting in the formation of 6-aminouracil and depletion of NO (28). Also, elevated UA was found to inhibit the production of NO in bovine aortic endothelial cells caused by the vascular endothelial growth factor (27). This evidence proves that hyperuricemia-induced vascular insufficiency can be achieved by reducing NO production. Besides, the angiotensin system is also believed to involve in UA-induced NO production reduction. According to earlier evidence, UA activates the renin-angiotensin system and inhibits NO production by downregulating NOS1 expression.

UA can also indirectly affect NO production through classical inflammatory pathways. Different concentration of UA *in vitro* has been found to involve Ikbα phosphorylation via NF-kB activated inflammatory signaling pathway. This leads to the up-regulation of inducible NO synthase (iNOS) expression and excessive NO production, which subsequently contribute to the injury of cells (17).

Elevated blood pressure is a known risk factor for AF, and hypertension often coexists with AF. Whereas, hyperuricemia, as a risk factor of AF, can directly trigger the occurrence of AF or indirectly contribute to AF through a hypertension-induced mechanism. Mazzali et al. used oxonic acid (OA) to create a mild hyper-UA model *in vivo*. After a low-salt diet in the rats, they found that the diameter of the arterioles of the mice with hyperuricemia was shortened and the blood pressure increased significantly (22). What's more, vascular hypertension can cause an increase in cardiac preload, resulting in left atrial structural remodeling (26). Studies have shown that after entering into cells through an organic anion transport, UA stimulates the proliferation of vascular smooth muscle cells. The changes in the vascular muscle cells may increase the thickness of the vascular wall, which could further result in cardiac afterload, and long-term atrial structural remodeling (29, 30), which could be the potential mechanism of AF.

### UA Participates in the Progress of Metabolic Syndrome-Induced AF

Experimental studies proposed that UA may have a causal role in metabolic syndrome (MetS) and obesity (22, 31). Earlier evidence pointed out that UA enters the cell through UA transporters, and intracellular UA increases the activity of xanthine oxidase (XO) and NADPH oxidase (NOX) (18). As a result, these activities promote the formation of superoxide. These common UA transporters include URATv1, ABCG2, MRP4, and MCT9 (16). A piece of evidence revealed, soluble UA provokes an increase in NOX activity in differentiated 3T3-L1 adipocytes by promoting the action of URATv1. The NOX activity *per se* is a cytoplasmic enzyme consisting of at least one catalytic transmembrane-spanning NOX subunit, which produces ROS by transferring electrons from NADPH to molecular oxygen. In addition, the reduction of bioavailability can result in the down-regulation of NO and an increase in protein nitrosylation and lipid oxidation (14, 19). Consequently, the formation of downstream superoxide-dependent ROS is increased, which leads to the up-regulation of monocyte chemotactic protein (MCP-1). These pathophysiological alterations can eventually lead to obesity-related low-grade inflammation, metabolic syndrome, and cardiovascular diseases (32).

### The Role of Oxidative Stress on the Progress of Elevated UA-Induced AF

A substantial body of evidence suggests that oxidative stress plays a key role in the pathophysiology of AF. However, the molecular pathways of this pathologic process are complex. Therefore, oxidative stress and its modulation in AF require the development of strategies that target specific sources of ROS implicated in atrial remodeling (33). XO is deemed to be a key enzyme in UA metabolism, which is also a critical source of reactive oxygen species (ROS), free radicals responsible for oxidative damage (34) in cardiovascular diseases (35). A study that involved a histochemical staining technique based on the reduction of nitro blue tetrazolium to formazan by superoxide radical also revealed the presence of XO activity in human hearts (36). Moreover, an analysis of the correlation between maximal oxygen and UA level in patients with chronic heart failure reflects the impairment of oxidative metabolism (37). Autonomic nervous system activation can induce significant and heterogeneous changes of atrial electrophysiology and induce atrial tachyarrhythmias, including atrial tachycardia and AF (38). Recently, some researchers showed that a continuous 4 weeks inhibition of XO in infarcted rats down-regulated sympathetic innervation (39). This suggests that UA involves in sympathetic nerve activity via sympathetic innervation probably through a superoxide-dependent pathway, which eventually contributes to arrhythmia.

Cell experiments have been conducted to reveal the effect of UA on cardiac remolding by stimulating the vascular Renin-Angiotensin System (RAS) (40). The study demonstrated that UA stimulates vascular smooth muscle cell proliferation and oxidative stress via the vascular renin-angiotensin system. Landmesser et al. have proved that angiotensin II induces the increased activity of NOX and XO, and eventually causes oxidative damage (41). An experimental test by Corry et al. also found that the mRNA and intracellular protein of angiotensin II were upgraded after 48 h of UA stimulation of vascular smooth muscle cells, and this effect was inhibited after the use of losartan and captopril (40). Moreover, increased oxidative stress levels that result in the upregulation of hydrogen peroxide and 8-isoprostatin was slowed down by losartan, captopril, and PD 98059 (a mitogen-activated protein (MAP) kinase inhibitor) treatment, suggesting UA causes vascular dysfunction through the angiotensin system.

Note, elevated UA levels cannot only increase the risk of myocardial oxidative damage through activating RAS but also lead to cellular damage by activating other pathways. For instance, a shred of evidence has shown that high UA can promote the up-regulation of NOX4 expression in renal proximal tubule cells and result in an increase in ROS production via activating P38 and ERK1/2 phosphorylation. Such ROS production through activation of P38 and ERK1/2 phosphorylation can further inhibit PI3K and Akt activation, and unbalance Bax/Bcl-2 equilibrium, which could eventually contribute to increased apoptosis and decreased cell activity (42).

Hyperuricemia can also cause oxidative damage and inhibit cardiomyocyte activity. Similar results were obtained *in vivo*, consistent with those obtained *in vitro*. The phosphorylation of ERK and P38 was up-regulated in mice with acute hyperuricemia model (15). Another experimental study endorsed hyperuricemia-induced oxidative stress in cardiomyocytes to further progress to myocardial structural remodeling (43).

Plasma urate level is directly regulated by a voltage-driven urate efflux transporter (URATv1) in humans (44). Recently, researchers have found that UA in the blood can promote the formation of ROS in atrial myocytes through UA transporters. In their study, they claimed that ROS activates ERK pathways and regulates the mRNA and protein expression of KV1.5, Nav1.5, and HERG channels. These researchers also demonstrated that hyperuricemia induces atrial electrical remodeling by activating URATv1. And after using URATV1 inhibitors, the expression of related sodium and potassium channel proteins and mRNA decreased. At the same time, this electrical remodeling effect was also inhibited by NOX inhibitors, apocynin, and antioxidant N-acetylcysteine (18). A recent experimental study examined the relationship between oxidative stress and atrial electrical and structural remodeling, and the beneficial effects of XO inhibitor allopurinol in alloxan-induced diabetes mellitus rabbits. The study found that allopurinol attenuated atrial structural and electrical remodeling and suppresses AF vulnerability. These protective effects of allopurinol were highly associated with reductions in ROS formation and atrial fibrosis-related factors and abnormal calcium homeostasis (45). Allopurinol can improve atrial electrical remodeling by inhibiting CaMKII activity and protein expression of Na+/Ca2+ exchanger in diabetic rats (46). In aggregate, these findings indicate that oxidative stress is involved in atrial electrical remodeling caused by hyperuricemia, and XO inhibition can reduce oxidative stress and ameliorate atrial electrical remodeling.

### The Role of Inflammation on the Progress of Elevated UA Induced-AF

Systemic inflammation, in particular, has been connected to endocardial inflammation of the endothelium, as well as atrial remodeling (47). Experimental animal models and human studies have verified that several major signaling pathways, including the renin-angiotensin system and inflammation, are involved in AF (48). Earlier evidence documented that hyperuricemia is responsible, at least in part, for the increased cytokine production. For example, Netea et al. explored the role of hyperuricemia in increased cytokine production after lipopolysaccharide challenge in neutropenic mice, and hyperuricemia induced by repeated administrations of uric acid in normal mice led to an increased TNF production after lipopolysaccharide (49). In general, an increase in cytokine is an indicator of systemic inflammation. In 2016, Chen et al. found that a combination of Na^+^ and UA can trigger an overproduction of TNF, IL-1, IL-6, and KC (50). In addition, some cytokine was reported to participate in the process of electrical and structural remolding of the heart, such as TNFα (51), and IL-1β (52).

In response to oxidative stress, activation of the inflammation signaling pathway NF-KB is reported to have direct effects on ion channel promoter regions, transcription factor expression levels, or mRNA splicing, which are responsible for the occurrence of AF. Acting on cultured tubular epithelial cells through the UA transporters, UA activates NF-KB signaling pathways and induces the expression of inflammatory factors and chemokines, including TNF-α, MCP-1, and RANTES (53). Moreover, UA activates the transcription factors nuclear factor-kappaB and activator protein-1, as well as the MAPK signaling molecules ERK p44/42 and p38, and increased cyclooxygenase-2 (COX-2) mRNA expression (24). Notably, UA-induced MCP-1 expression at 24 h was suppressed following the inhibition of p38, ERK 44/42, or COX-2, implicating these pathways in response to UA. Also, the ability of both diphenyleneionium (antioxidants) and n-acetyl-cysteine to obstruct UA-induced MCP-1 production suggested the involvement of intracellular redox pathways. Therefore, it is worthy to conclude that UA regulates critical proinflammatory pathways.

The effects of UA on human umbilical vein endothelial cells (HUVEC) and human vascular smooth muscle cells have been reported to produce a certain degree of vascular inflammation and vascular remodeling, mainly in the up-regulation of the expression of C-reactive protein, one of the independent risk factors for cardiovascular diseases and an important marker of inflammation (54). Another study showed that soluble UA, at physiologic concentrations, has profound effects on human vascular cells. The study reported that UA alters the proliferation/migration and NO release of human vascular cells, mediated by the expression of CRP (23). This suggests UA induced inflammation cause vascular dysfunction.

When UA stimulates endothelial cells, it induces the acetylation of an intracellular high-mobility group box-1 protein (HMGB1) through calcium mobilization and MERK/ERK pathway. A study that involved treatment of HUVEC with UA resulted in increased HMGB1 mRNA expression and acetylation of nuclear HMGB1 (55). UA after ischemia-reperfusion injury mediates the acetylation and release of HMGB1 from endothelial cells by the MEK/Erk pathway, calcium mobilization, and activation of Toll-like receptor-4. Once released, HMGB1 *per se* promotes its cellular release and acts as an autocrine and paracrine to activate both proinflammatory and pro-reparative mediators (16). Besides, UA has the ability to stimulate pro-inflammatory effect in vascular smooth muscle cells to produce MCP-1 at transcriptional levels and protein expressions. Also, UA could cause necrosis of human mesangial cells at an ecological concentration, but UA could increase significantly at 8 mg/dl concentration and then increase the expression of COX-2 and PGE-2 to promote inflammation (25).

### The Role of Fibrosis on the Process of Elevated UA-Induced AF

Atrial fibrosis produces heterogeneous pathways of slow conduction and atrial dilatation. In male adult Sprague Dawley rats, abnormal morphology of atrial myocytes, apoptosis, and atrial fibrosis were observed in hyperuricemic rats compared to the control. The study demonstrated that apoptosis and fibrosis of atria were partly mediated by B-cell lymphoma 2-extra-large (Bax), caspase-3, α-smooth muscle actin, and TGF-β1. Also, the study reported that uric acid significantly induced primary rat cardiomyocyte apoptosis and fibrosis *in vitro* and AF induced by hyperuricemic rats occurred primarily via induction of atrial remodeling (56). Also, another study supports that UA can induce cell proliferation and endothelin-1 gene expression in rats with myocardial fibroblasts. Notably, the researchers were able to reverse the myocardial fibroblasts by increasing NADPH oxidase activity, ROS generation, ERK phosphorylation, and activator protein-1(AP-1)-mediated replacement activity (21). Thus, UA plays an important role in the pathogenesis of cardiac fibroblasts. Other researchers who were used the OA to create a mild hyper UA model *in vivo*, after a low-salt diet in the mice, found the shortened diameter of the arterioles in the hyperuricemia group and a significant increase in blood pressure (57). Likewise, some researchers reported an increase in cardiac afterload, interstitial fibrosis, and collagen deposition in the hyperuricemia mice compared with those in the control group (58). Importantly, another study draws a similar conclusion in mice fed a Western diet. In assessing the role of Western diet-induced increases in uric acid, the researchers found that increased cardiomyocyte hypertrophy, interstitial fibrosis, myocardial oxidative stress, and impaired diastolic relaxation. Further, the Western diet enhanced the profibrotic transforming growth factor-β1/Smad2/3 signaling pathway, activation of the S6 kinase-1 growth pathway, and macrophage proinflammatory polarization. Importantly, all these Western diet-induced pathophysiological alterations were improved with allopurinol treatment (59). These results suggest increased production of UA promotes cardiomyocyte hypertrophy, oxidative stress, and inflammation that result in myocardial fibrosis and associated impaired diastolic relaxation.

### The Role of Apoptosis and Immunity on the Progress of the AF

A significant association has been reported between UA and cell apoptosis. Of note, Bcl2 family proteins are key regulators of apoptosis cell death. An experimental study suggested a high concentration of UA downregulates B-cell lymphoma 2 (Bcl-2) expression in pancreatic β-cells (17). This could substantially lead to the imbalance of Bax/Bcl-2. In another recent experimental study, Yan et al. demonstrated, uric acid induces cardiomyocyte apoptosis via activation of calpain-1 and endoplasmic reticulum stress (ERS) (60). The study extended that Calpain-1 expression was significantly increased after oxonic acid (OA) gavage administration for 16 weeks and p-PERK, GRP78, and CHOP expression was increased in H9c2 cardiomyocytes, suggesting hyperuricemia induced ERS activation. Another study demonstrated that elevated UA promoted ROS-induced tubular cell apoptosis by upregulating Nox4 expression in HK-2 cells that were used as a human proximal tubular cell model. The results of the study showed that treatment with UA reduced HK-2 cell viability and enhanced apoptosis in a dose-dependent manner. This was consistent with Nox4 upregulation as well as ROS overproduction, which resulted in Bax/Bcl-2 imbalance in HK-2 cells. Interestingly, inhibition of Nox4 with DPI prevented UA-induced cell apoptosis (14). Therefore, UA may involve in atrial fibrosis induced AF.

Immune cells may play a vital role in the immunological pathogenesis of AF (61), and UA has been identified as one of the endogenous adjuvants that can augment immune responses to particulate antigens (62). UA alerts the immune system to dying cells by stimulating dendritic cell maturation and activation (63). The study demonstrated that UA increases the expression of dendritic cell maturation markers, including CD80 and CD86. Also, Webb et al. demonstrated that UA can directly activate T-cells in the absence of antigen presentation (64). UA release leads to antigen-independent T-cell stimulation and provides an adjuvant effect for autoantigens released from apoptotic cells. It is reported, UA could lower the threshold for T-cell activation and potentially facilitate the break of peripheral T-cell tolerance (64). When UA stimulates endothelial cells, it activates toll receptor four, which eventually leads to increased expression and secretion of angiotensin II and stimulation of NF-κB inflammatory channels (16).

## Clinical Evidence: Hyperuricemia and Atrial Fibrillation

Epidemiological evidence suggests that the prevalence of hyperuricemia and AF are on the rise in many corners of the world. According to a community-based study that involved the elderly population, high UA population (SUA > 416 μmol/L in men and >357 μmol/L in women) had a higher risk of AF (OR: 2.080, 95% CI: 1.103–4.202; *P* < 0.001) (65). A single centered observational study by Mantovani et al. also concluded that hyperuricemia is independently associated with increased prevalence of AF [odds ratio (OR): 3.41, 95 % confidence interval (CI): 2.19–5.32; *p* < 0.001] in patients with type 2 diabetes after adjusted for multiple confounding factors (66). Also, a cohort study from Taiwan reported similar findings (67). Over a mean follow-up of 6.3 years, Chao et al. reported that individuals with a history of one or more episodes of gouty arthritis had a higher risk of AF (2.1 vs. 1.7% in controls, *P* < 0.001), even after adjustment for age and gender (67). Another study also showed a positive and independent association between UA and AF in coronary heart disease (CHD) events complicated with chronic comorbidity (68). This evidence from the epidemiological studies may explain the tremendous influence of the chronic disease comorbidity in the association between hyperuricemia and AF. Table 2 summarizes related studies regarding the elevated UA and risk of AF.

**Table 2:** The impact of hyperuricemia on the risk of atrial fibrillation.

**References**	**Study design**	**Research population**	**Number of patients with AF**	**Number of population**	**Main finding**
Sun et al. (69)	Cross-sectional	People from rural regions	139	11,956	Hyperuricemia is closely related to the increased prevalence of AF with the OR of 1.94
Mantovani et al. (66)	Cross-sectional	T2DM inpatients	91	867	T2DM patients with hyperuricemia had a greater likelihood to have AF than patients with normal uric acid level (OR: 3.41)
Kuwabara et al. (70)	Randomized control	General population	291	90,143	AF groups (OR: 1.35) have an significantly higher SUA level and hyperuricemia (OR: 1.73) was a significantly independent competing risk factor for AF
Liu et al. (61)	Cross-sectional	General population	55	1,056	Hyperuricemia (OR: 1.69) was an independent predictor for left atrial thrombus/spontaneous echo contrast in non-valvular AF patients
Lin et al. (71)	Cross-sectional	Residents of community	144	11,488	Elevated SUA level (OR: 2.19) is independently with the increased risk of AF
Zhang et al. (11)	Cohort	Individuals	6,831	527,908	The highest (HR:1.9) and the intermediate level (1.36) of UA significantly increase the risk of AF after adjusted for traditional risk factors
Li et al. (10)	Cohort	General population	871	123,238	High SUA level (HR:1.91) and the augment in SUA increases the occurrence of AF
Kawasoe et al. (72)	Cohort	General population	647	111,566	High level of baseline SUA (HR:1.74) was closely related with a higher incidence of AF in women.
Hong et al. (73)	Mendelian Random	Inpatients	633	4,166	Hyperuricemia gene rs1165196 (OR: 0.21) was causally associated with AF
Zhang et al. (74)	Meta-analysis	General population	N/A	426,159	Hyperuricemia (RR: 1.49) was significantly associated with increased risk of AF

*T2DM, Type 2 diabetes; AF, Atrial fibrillation; SUA, serum uric acid; CHD, Coronary heart disease; OR, odds ratio; HR, hazard ratio; RR, relative risk*.

Different treatment modalities have shown different results. A cohort study tested the use of allopurinol and the risk of atrial fibrillation in the elderly in 8,569 beneficiaries using Medicare data. After adjustment for age, sex, race, Charlson–Romano comorbidity index, and use of statins, diuretics, ACE inhibitors, and β-blockers, the use of allopurinol for 6 months or more was associated with a reduced risk of incident AF in the elderly (HR: 0.83; 95% CI: 0.74–0.93) (75). On the other hand, a meta-analysis of two retrospectives and two prospective cohort studies showed that elevated UA was not associated with an increased risk of AF recurrence after catheter ablation (76). However, the meta-analysis was criticized for large heterogeneity regarding AF type, ablation technique, and follow-up duration. Also, the meta-analysis included a small number of studies. In summary, clinical and epidemiological evidence showed that gender, aging of the population, CVD comorbidity, and allopurinol drugs seem to affect the prevalence of AF.

Colchicine, an anti-inflammatory drug that is used for a wide range of inflammatory diseases also associated with decreasing AF risk. A meta-analysis that included 17 prospective controlled randomized studies with 2082 patients that received colchicine and 1982 controls with an average follow-up duration of 12 months reported that treatment with colchicine reduced the recurrence of atrial fibrillation significantly in patients after cardiac surgery or pulmonary vein isolation (OR: 0.54, 95% CI: 0.41-0.7; *P* = 0.001) (77). However, a double-blind, placebo-controlled, randomized clinical trial among 360 patients undergoing cardiac surgery indicated that perioperative use of colchicine compared with placebo reduced the incidence of the postpericardiotomy syndrome but not of postoperative AF or postoperative pericardial/pleural effusion (78). Further RCTs are required to determine if AF events are lowered with colchicine.

### Relationship Between Uric Acid Level and Atrial Fibrillation

Several studies have demonstrated the association between hyperuricemia and the development of AF. These studies that show a clear association between hyperuricemia and AF raise the question: how much elevation in UA concentration will significantly increase the risk of AF? Kuwabara et al. found that UA concentration was significantly higher in patients with AF than in non-AF (OR: 2.75; 95% CI: 1.22–1.50; *P* < 0.05) after adjusting for traditional risk factors such as hypertension, diabetes, nephropathy, and lipid metabolism disorders (70). And the incidence of AF increases when UA levels reach a certain limit. For instance, the first dose-response meta-analysis regarding the relationship between elevated UA concentration and the incidence of AF reported that both the uppermost [Relative risk (RR): 1.9, 95% CI: 1.64–2.23; *I*^2^ = 0%] and medial (RR: 1.36, 95% CI: 1.16–1.59; *I*^2^ = 36%) level of serum UA were associated with increased risks of AF in comparison to patients with the lowest level of serum UA (79). The Kailuan cohort study has also reported similar findings. After repeated measurements of UA concentrations, patients with high levels of both serum UA had a substantially higher risk of AF. Most importantly, the study has denoted that the first rise in UA concentration is sufficient to increase the risk of AF in both men and women (male UA baseline level was >6.5 mg/dL, and female UA baseline level was >4.9 mg/dL) (10). This evidence is sufficient that hyperuricemia is associated with an increased risk of AF in patients with or without other chronic conditions.

Previous studies reported a positive association between UA levels and left atrial diameter in patients with hypertension. A study that included 451 hypertensive patients demonstrated an independent association between increased serum UA levels and AF (80). Also, a single centered retrospective data from Northeast China showed that SUA levels and left atrial diameter (LAD) were associated with AF in patients with hypertension, and the risk of AF associated with LAD increases among those with hyperuricemia (Hidru et al.). It should be noted that enlarged left atrial diameter is a conventional marker of atrial structural remodeling (81). Therefore, UA may participate in the pathophysiology of AF in patients with hypertension.

UA is an independent risk factor for MetS (82) and patients with MetS are considered to be at a higher risk of developing AF (83). A prospective, observational study that enrolled 843 AF patients (mean age, 62.5 ± 12.1 years, 38.6% female) without overt coronary artery disease reported a significant risk of major adverse cardiovascular events, including myocardial infarction, coronary revascularization, and cardiac death (84). The study suggested that the prevention and treatment of MetS may reduce the burden of non-thromboembolic complications in AF. Since AF often associates with diabetes, hypertension, and obesity, it is possible that convergence of multiple risk factors could potentiate AF risk. As such, understanding the link among MetS, UA, AF, and non-thromboembolic MACE is imperative for investigating the possible mechanism and devising effective preventive strategies.

### The Effect of Gender in the Link Between Uric Acid and Atrial Fibrillation

The reports on the effect of hyperuricemia on AF incidence have tremendous discrepancies. Epidemiological evidence suggested that hyperuricemia was more prevalent in males than females (7.9 vs. 4.9%) (85). Similarly, a cross-sectional study in three rural regions of China revealed a positive relationship between hyperuricemia and AF in men, but not in women (86). In contrast, a recent large cross-sectional study that includes urban and rural residents revealed a significant association between high UA levels and the prevalence of AF, especially in females (10). Also, a survey of healthy adults found that hyperuricemia is broadly correlated to the occurrence of cardiovascular diseases in females (87). And earlier in 2012, a Japanese study of 7,155 patients reported that hyperuricemia significantly increased the prevalence of crude AF, but after adjusting for all cardiovascular risk factors, such independent correlations were only confirmed in women (OR: 1.888, 95% CI: 1.278–2.790; *P* < 0.05) but not in men (OR: 1.176, 95% CI: 0.935–1.478; *P* > 0.05) (88). Similarly, prospective follow-up studies confirmed that women with high UA are more likely to develop AF (89), although the number of women with high UA is much lower than that of men (90). Recently, a large prospective study of 15,737 participants (52% women), tested the established and novel risk factors for atrial fibrillation in women compared with men, over 20 years follow-up. Women showed a stronger relationship between UA and AF (91).

### The Causal Relationship Between UA and AF

Ample evidence is available from a large number of clinical studies to reach a consensus that hyperuricemia is an independent risk factor for AF. However, whether UA causes AF required explicit evidence. A human study has confirmed the association between UA and concentration levels of cytokine including C-reactive protein (CRP), IL-6, and TNF-α and IL-1β, especially in women (26). This evidence explains that UA induced inflammation involve pathophysiological alteration either directly or indirectly. A previous Mendelian randomization analysis indicated that a genetic causal relation between elevated UA level and adverse cardiovascular outcomes, such as sudden cardiac death (92). Hong et al. designed a genomic study on the susceptibility of AF associated with UA using 9 selected single nucleotide polymorphism (SNPs) and found that the SNP rs1165196 on SLC17A1 (F-statistics = 208.34, 0.18 mg/mL per allele change; *P* < 0.001) and weighted genetic risk score (wGRS) (F-statistics = 222.26, 0.20 mg/mL per 1 SD change; *P* < 0.001) were significantly associated with increased UA levels. The mendelian randomized analysis was causally associated with rs1165196 (OR: 0.21, 95% CI: 0.06–0.75; *P* = 0.017), but not with wGRS (OR: 1.07, 95% CI: 0.57–2.01; *P* = 0.832) (73), confirming that the UA was independently associated with the AF risk.

## Conclusion

Existing studies strongly suggest that hyperuricemia is independently associated with the increasing incidence of AF. Epidemiological and clinical studies highlight a close association between various conditions including hypertension, metabolic syndrome, DM, and other CVD comorbidities and increased risk of AF. Therefore, the mechanistic links between UA and AF are complex with several underlying diseases and conditions. Experimental and clinical data indicate that UA is implicated in the pathophysiology of AF via activation of inflammation, oxidative stress, and fibrosis induced atrial remodeling. Briefly, atrial remodeling involves electrophysiological and structural abnormalities that promote the development of UA induced AF. Also, UA induced AF activates apoptosis and immune system. There is still a need for further investigation to obtain a more comprehensive understanding of the role of UA in the pathophysiology of AF.

## Author Contributions

YD and FL were contributed to literature researches, data collection, and were involved in the draft of the manuscript. XY and YX were contributed to the coordination and designing of the review and writing of the final draft of the manuscript. All authors have read and approved the final manuscript.

## Conflict of Interest

The authors declare that the research was conducted in the absence of any commercial or financial relationships that could be construed as a potential conflict of interest.

## References

[1] Benjamin EJ Muntner P Alonso A Bittencourt MS Callaway CW Carson AP. Heart disease and stroke statistics-2019 update: a report from the american heart association. Circulation. (2019) 139:e56–28. 10.1161/CIR.0000000000000659 30700139

[2] Staerk L Sherer JA Ko D Benjamin EJ Helm RH. Atrial fibrillation: epidemiology, pathophysiology, and clinical outcomes. Circ Res. (2017) 120:1501–17. 10.1161/CIRCRESAHA.117.309732 28450367 PMC5500874

[3] Kornej J Borschel CS Benjamin EJ Schnabel RB. Epidemiology of atrial fibrillation in the 21st century: novel methods and new insights. Circ Res. (2020) 127:4–20. 10.1161/CIRCRESAHA.120.316340 32716709 PMC7577553

[4] Hart RG Pearce LA Aguilar MI. Meta-analysis: antithrombotic therapy to prevent stroke in patients who have nonvalvular atrial fibrillation. Ann Intern Med. (2007) 146:857–67. 10.7326/0003-4819-146-12-200706190-00007 17577005

[5] Virdis A Masi S Casiglia E Tikhonoff V Cicero AFG Ungar A. Identification of the uric acid thresholds predicting an increased total and cardiovascular mortality over 20 years. Hypertension. (2020) 75:302–8. 10.1161/HYPERTENSIONAHA.119.13643 31813345

[6] Lip GY Laroche C Ioachim PM Rasmussen LH Vitali-Serdoz L Petrescu L. Prognosis and treatment of atrial fibrillation patients by European cardiologists: one year follow-up of the EURObservational Research Programme-Atrial Fibrillation General Registry Pilot Phase (EORP-AF Pilot registry). Eur Heart J. (2014) 35:3365–76. 10.1093/eurheartj/ehu374 25176940

[7] Liu Y. Hyperuricemia and risk of atrial fibrillation. J Atr Fibrillation. (2014) 6:967. 10.4022/jafib.967 27957037 PMC4956125

[8] Harada M Van Wagoner DR Nattel S. Role of inflammation in atrial fibrillation pathophysiology and management. Circ J. (2015) 79:495–502. 10.1253/circj.CJ-15-0138 25746525 PMC4457364

[9] Feig DI Kang DH Johnson RJ. Uric acid and cardiovascular risk. N Engl J Med. (2008) 359:1811–21. 10.1056/NEJMra0800885 18946066 PMC2684330

[10] Li S Cheng J Cui L Gurol ME Bhatt DL Fonarow GC. Cohort Study of repeated measurements of serum urate and risk of incident atrial fibrillation. J Am Heart Assoc. (2019) 8:e012020. 10.1161/JAHA.119.012020 31213103 PMC6662349

[11] Zhang J Zheng R Li H Guo J. Serum uric acid and incident atrial fibrillation: a systematic review and dose-response meta-analysis. Clin Exp Pharmacol Physiol. (2020) 47:1774–82. 10.1111/1440-1681.13374 32621546

[12] Kang DH Ha SK. Uric acid puzzle: dual role as anti-oxidantand pro-oxidant. Electrolyte Blood Press. (2014) 12:1–6. 10.5049/EBP.2014.12.1.1 25061467 PMC4105384

[13] Yu W Cheng JD. Uric acid and cardiovascular disease: an update from molecular mechanism to clinical perspective. Front Pharmacol. (2020) 11:582680. 10.3389/fphar.2020.582680 33304270 PMC7701250

[14] Li Z Sheng Y Liu C Li K Huang X Huang J. Nox4 has a crucial role in uric acidinduced oxidative stress and apoptosis in renal tubular cells. Mol Med Rep. (2016) 13:4343–8. 10.3892/mmr.2016.5083 27052425

[15] Li Z Shen Y Chen Y Zhang G Cheng J Wang W. High uric acid inhibits cardiomyocyte viability through the ERK/P38 pathway via oxidative stress. Cell Physiol Biochem. (2018) 45:1156–64. 10.1159/000487356 29444507

[16] Rabadi MM Kuo MC Ghaly T Rabadi SM Weber M Goligorsky MS. Interaction between uric acid and HMGB1 translocation and release from endothelial cells. Am J Physiol Renal Physiol. (2012) 302:F730–41. 10.1152/ajprenal.00520.2011 22189943 PMC3311321

[17] Jia L Xing J Ding Y Shen Y Shi X Ren W. Hyperuricemia causes pancreatic beta-cell death and dysfunction through NF-kappaB signaling pathway. PLoS ONE. (2013) 8:e78284. 10.1371/journal.pone.0078284 24205181 PMC3808354

[18] Maharani N Ting YK Cheng J Hasegawa A Kurata Y Li P. Molecular mechanisms underlying urate-induced enhancement of Kv1.5 channel expression in HL-1 atrial myocytes. Circ J. (2015) 79:2659–68. 10.1253/circj.CJ-15-0416 26477273

[19] Sautin YY Nakagawa T Zharikov S Johnson RJ. Adverse effects of the classic antioxidant uric acid in adipocytes: NADPH oxidase-mediated oxidative/nitrosative stress. Am J Physiol Cell Physiol. (2007) 293:C584–96. 10.1152/ajpcell.00600.2006 17428837

[20] Zhi L Yuzhang Z Tianliang H Hisatome I Yamamoto T Jidong C. High uric acid induces insulin resistance in cardiomyocytes *in vitro* and *in vivo*. PLoS ONE. (2016) 11:e0147737. 10.1371/journal.pone.0147737 26836389 PMC4737875

[21] Cheng TH Lin JW Chao HH Chen YL Chen CH Chan P. Uric acid activates extracellular signal-regulated kinases and thereafter endothelin-1 expression in rat cardiac fibroblasts. Int J Cardiol. (2010) 139:42–9. 10.1016/j.ijcard.2008.09.004 18945502

[22] Mazzali M Hughes J Kim YG Jefferson JA Kang DH Gordon KL. Elevated uric acid increases blood pressure in the rat by a novel crystal-independent mechanism. Hypertension. (2001) 38:1101–6. 10.1161/hy1101.092839 11711505

[23] Kang DH Park SK Lee IK Johnson RJ. Uric acid-induced C-reactive protein expression: implication on cell proliferation and nitric oxide production of human vascular cells. J Am Soc Nephrol. (2005) 16:3553–62. 10.1681/ASN.2005050572 16251237

[24] Kanellis J Watanabe S Li JH Kang DH Li P Nakagawa T. Uric acid stimulates monocyte chemoattractant protein-1 production in vascular smooth muscle cells via mitogen-activated protein kinase and cyclooxygenase-2. Hypertension. (2003) 41:1287–93. 10.1161/01.HYP.0000072820.07472.3B 12743010

[25] Convento MS Pessoa E Dalboni MA Borges FT Schor N. Pro-inflammatory and oxidative effects of noncrystalline uric acid in human mesangial cells: contribution to hyperuricemic glomerular damage. Urol Res. (2011) 39:21–7. 10.1007/s00240-010-0282-5 20524111

[26] Zharikov S Krotova K Hu H Baylis C Johnson RJ Block ER. Uric acid decreases NO production and increases arginase activity in cultured pulmonary artery endothelial cells. Am J Physiol Cell Physiol. (2008) 295:C1183–90. 10.1152/ajpcell.00075.2008 18784379 PMC2584985

[27] Khosla UM Zharikov S Finch JL Nakagawa T Roncal C Mu W. Hyperuricemia induces endothelial dysfunction. Kidney Int. (2005) 67:1739–42. 10.1111/j.1523-1755.2005.00273.x 15840020

[28] Gersch C Palii SP Kim KM Angerhofer A Johnson RJ Henderson GN. Inactivation of nitric oxide by uric acid. Nucleosides Nucleotides Nucleic Acids. (2008) 27:967–78. 10.1080/15257770802257952 18696365 PMC2701227

[29] Rao GN Corson MA Berk BC. Uric acid stimulates vascular smooth muscle cell proliferation by increasing platelet-derived growth factor A-chain expression. J Biol Chem. (1991) 266:8604–8. 2022672

[30] Kang DH Han L Ouyang X Kahn AM Kanellis J Li P. Uric acid causes vascular smooth muscle cell proliferation by entering cells via a functional urate transporter. Am J Nephrol. (2005) 25:425–33. 10.1159/000087713 16113518

[31] Hikita M Ohno I Mori Y Ichida K Yokose T Hosoya T. Relationship between hyperuricemia and body fat distribution. Intern Med. (2007) 46:1353–8. 10.2169/internalmedicine.46.0045 17827832

[32] Baldwin W McRae S Marek G Wymer D Pannu V Baylis C. Hyperuricemia as a mediator of the proinflammatory endocrine imbalance in the adipose tissue in a murine model of the metabolic syndrome. Diabetes. (2011) 60:1258–69. 10.2337/db10-0916 21346177 PMC3064099

[33] Korantzopoulos P Letsas K Fragakis N Tse G Liu T. Oxidative stress and atrial fibrillation: an update. Free Radic Res. (2018) 52:1199–209. 10.1080/10715762.2018.1500696 30003814

[34] Doehner W Landmesser U. Xanthine oxidase and uric acid in cardiovascular disease: clinical impact and therapeutic options. Semin Nephrol. (2011) 31:433–40. 10.1016/j.semnephrol.2011.08.007 22000650

[35] Glantzounis GK Tsimoyiannis EC Kappas AM Galaris DA. Uric acid and oxidative stress. Curr Pharm Des. (2005) 11:4145–51. 10.2174/138161205774913255 16375736

[36] MacGowan SW Regan MC Malone C Sharkey O Young L Gorey TF. Superoxide radical and xanthine oxidoreductase activity in the human heart during cardiac operations. Ann Thorac Surg. (1995) 60:1289–93. 10.1016/0003-4975(95)00616-S 8526614

[37] Leyva F Anker S Swan JW Godsland IF Wingrove CS Chua TP. Serum uric acid as an index of impaired oxidative metabolism in chronic heart failure. Eur Heart J. (1997) 18:858–65. 10.1093/oxfordjournals.eurheartj.a015352 9152657

[38] Chen PS Chen LS Fishbein MC Lin SF Nattel S. Role of the autonomic nervous system in atrial fibrillation: pathophysiology and therapy. Circ Res. (2014) 114:1500–15. 10.1161/CIRCRESAHA.114.303772 24763467 PMC4043633

[39] Lee TM Lin SZ Chang NC. Effects of urate-lowering agents on arrhythmia vulnerability in post-infarcted rat hearts. J Pharmacol Sci. (2016) 131:28–36. 10.1016/j.jphs.2016.03.009 27129614

[40] Corry DB Eslami P Yamamoto K Nyby MD Makino H Tuck ML. Uric acid stimulates vascular smooth muscle cell proliferation and oxidative stress via the vascular renin-angiotensin system. J Hypertens. (2008) 26:269–75. 10.1097/HJH.0b013e3282f240bf 18192841

[41] Landmesser U Spiekermann S Preuss C Sorrentino S Fischer D Manes C. Angiotensin II induces endothelial xanthine oxidase activation: role for endothelial dysfunction in patients with coronary disease. Arterioscler Thromb Vasc Biol. (2007) 27:943–8. 10.1161/01.ATV.0000258415.32883.bf 17234726

[42] Han HJ Lim MJ Lee YJ Lee JH Yang IS Taub M. Uric acid inhibits renal proximal tubule cell proliferation via at least two signaling pathways involving PKC, MAPK, cPLA2, and NF-kappaB. Am J Physiol Renal Physiol. (2007) 292:F373–81. 10.1152/ajprenal.00104.2006 16985215

[43] Chen CC Hsu YJ Lee TM. Impact of elevated uric acid on ventricular remodeling in infarcted rats with experimental hyperuricemia. Am J Physiol Heart Circ Physiol. (2011) 301:H1107–17. 10.1152/ajpheart.01071.2010 21622823

[44] Anzai N Ichida K Jutabha P Kimura T Babu E Jin CJ. Plasma urate level is directly regulated by a voltage-driven urate efflux transporter URATv1 (SLC2A9) in humans. J Biol Chem. (2008) 283:26834–8. 10.1074/jbc.C800156200 18701466

[45] Yang Y Zhao J Qiu J Li J Liang X Zhang Z. (2018). Xanthine Oxidase Inhibitor Allopurinol Prevents Oxidative Stress-Mediated Atrial Remodeling in Alloxan-Induced Diabetes Mellitus Rabbits. J Am Heart Assoc 7(10). 10.1161/JAHA.118.008807 29720500 PMC6015332

[46] Yang Y He J Yuan M Tse G Zhang K Ma Z. Xanthine oxidase inhibitor allopurinol improves atrial electrical remodeling in diabetic rats by inhibiting CaMKII/NCX signaling. Life Sci. (2020) 259:118290. 10.1016/j.lfs.2020.118290 32822713

[47] Korantzopoulos P Letsas KP Tse G Fragakis N Goudis CA Liu T. Inflammation and atrial fibrillation: a comprehensive review. J Arrhythm. (2018) 34:394–401. 10.1002/joa3.12077 30167010 PMC6111477

[48] Gao G Dudley SCJr. Redox regulation, NF-kappaB, and atrial fibrillation. Antioxid Redox Signal. (2009) 11:2265–77. 10.1089/ARS.2009.2595 19309257 PMC2819799

[49] Netea MG Kullberg BJ Blok WL Netea RT van der Meer JW. The role of hyperuricemia in the increased cytokine production after lipopolysaccharide challenge in neutropenic mice. Blood. (1997) 89:577–82. 9002961

[50] Chen CJ Shi Y Hearn A Fitzgerald K Golenbock D Reed G. MyD88-dependent IL-1 receptor signaling is essential for gouty inflammation stimulated by monosodium urate crystals. J Clin Invest. (2006) 116:2262–71. 10.1172/JCI28075 16886064 PMC1523415

[51] Sawaya SE Rajawat YS Rami TG Szalai G Price RL Sivasubramanian N. Downregulation of connexin40 and increased prevalence of atrial arrhythmias in transgenic mice with cardiac-restricted overexpression of tumor necrosis factor. Am J Physiol Heart Circ Physiol. (2007) 292:H1561–7. 10.1152/ajpheart.00285.2006 17122196

[52] Wang CH Hu DY Tang CZ Wu MY Mei YQ Zhao JG. [Changes of interleukin-1beta and tumor necrosis factor-alpha of right atrial appendages in patients with rheumatic valvular disease complicated with chronic atrial fibrillation]. Zhonghua Xin Xue Guan Bing Za Zhi. (2005) 33:522–5. 16053785

[53] Zhou Y Fang L Jiang L Wen P Cao H He W. Uric acid induces renal inflammation via activating tubular NF-kappaB signaling pathway. PLoS ONE. (2012) 7:e39738. 10.1371/journal.pone.0039738 22761883 PMC3382585

[54] Ogita M Miyauchi K. C-reactive protein and cardiovascular disease. J Cardiol. (2016) 68:179. 10.1016/j.jjcc.2015.11.001 27264276

[55] Li J Wang S Bai J Yang XL Zhang YL Che YL. Novel role for the immunoproteasome subunit PSMB10 in angiotensin II-induced atrial fibrillation in mice. Hypertension. (2018) 71:866–76. 10.1161/HYPERTENSIONAHA.117.10390 29507100

[56] Wang D Sun L Zhang G Liu Y Liang Z Zhao J. Increased susceptibility of atrial fibrillation induced by hyperuricemia in rats: mechanisms and implications. Cardiovasc Toxicol. (2021) 21:192–205. 10.1007/s12012-020-09611-4 33099748

[57] Watanabe S Kang DH Feng L Nakagawa T Kanellis J Lan H. Uric acid, hominoid evolution, and the pathogenesis of salt-sensitivity. Hypertension. (2002) 40:355–60. 10.1161/01.hyp.0000028589.66335.aa 12215479

[58] Mazzali M Kim YG Suga S Gordon KL Kang DH Jefferson JA. Hyperuricemia exacerbates chronic cyclosporine nephropathy. Transplantation. (2001) 71:900–5. 10.1097/00007890-200104150-00014 11349724

[59] Jia G Habibi J Bostick BP Ma L DeMarco VG Aroor AR. Uric acid promotes left ventricular diastolic dysfunction in mice fed a Western diet. Hypertension. (2015) 65:531–9. 10.1161/HYPERTENSIONAHA.114.04737 25489061 PMC4370431

[60] Yan M Chen K He L Li S Huang D Li J. Uric acid induces cardiomyocyte apoptosis via activation of calpain-1 and endoplasmic reticulum stress. Cell Physiol Biochem. (2018) 45:2122–35. 10.1159/000488048 29533935

[61] Liu FZ Liao HT Lin WD Xue YM Zhan XZ Fang XH. Predictive effect of hyperuricemia on left atrial stasis in non-valvular atrial fibrillation patients. Int J Cardiol. (2018) 258:103–8. 10.1016/j.ijcard.2018.01.080 29467096

[62] Hu DE Moore AM Thomsen LL Brindle KM. Uric acid promotes tumor immune rejection. Cancer Res. (2004) 64:5059–62. 10.1158/0008-5472.CAN-04-1586 15289304

[63] Shi Y Evans JE Rock KL. Molecular identification of a danger signal that alerts the immune system to dying cells. Nature. (2003) 425:516–21. 10.1038/nature01991 14520412

[64] Webb R Jeffries M Sawalha AH. Uric acid directly promotes human T-cell activation. Am J Med Sci. (2009) 337:23–7. 10.1097/MAJ.0b013e31817727af 19057377

[65] Huang G Xu RH Xu JB Liu Y Liu ZH Xie X. Hyperuricemia is associated with atrial fibrillation prevalence in very elderly - a community based study in Chengdu, China. Sci Rep. (2018) 8:12403. 10.1038/s41598-018-30321-z 30120309 PMC6098088

[66] Mantovani A Rigolon R Pichiri I Pernigo M Bergamini C Zoppini G. Hyperuricemia is associated with an increased prevalence of atrial fibrillation in hospitalized patients with type 2 diabetes. J Endocrinol Invest. (2016) 39:159–67. 10.1007/s40618-015-0354-z 26178737

[67] Chao TF Hung CL Chen SJ Wang KL Chen TJ Lin YJ. The association between hyperuricemia, left atrial size and new-onset atrial fibrillation. Int J Cardiol. (2013) 168:4027–32. 10.1016/j.ijcard.2013.06.067 23871344

[68] Murakami N Tanno M Kokubu N Nishida J Nagano N Ohnishi H. Distinct risk factors of atrial fibrillation in patients with and without coronary artery disease: a cross-sectional analysis of the BOREAS-CAG Registry data. Open Heart. (2017) 4:e000573. 10.1136/openhrt-2016-000573 28123767 PMC5255559

[69] Sun GZ Guo L Wang J Ye N Wang XZ Sun YX. Association between hyperuricemia and atrial fibrillation in rural China: a cross-sectional study. BMC Cardiovasc Disord. (2015) 15:98. 10.1186/s12872-015-0089-y 26324443 PMC4553942

[70] Kuwabara M Niwa K Nishihara S Nishi Y Takahashi O Kario K. Hyperuricemia is an independent competing risk factor for atrial fibrillation. Int J Cardiol. (2017) 231:137–42. 10.1016/j.ijcard.2016.11.268 27871785

[71] Lin WD Deng H Guo P Liu FZ Chen RY Fang XH. High prevalence of hyperuricaemia and its impact on non-valvular atrial fibrillation: the cross-sectional Guangzhou (China) Heart Study. BMJ Open. (2019) 9:e028007. 10.1136/bmjopen-2018-028007 31147367 PMC6549638

[72] Kawasoe S Kubozono T Yoshifuku S Ojima S Miyata M Miyahara H. Uric acid level and new-onset atrial fibrillation in the japanese general population- longitudinal study. Circ J. (2018) 83:156–63. 10.1253/circj.CJ-18-0508 30393244

[73] Hong M Park JW Yang PS Hwang I Kim TH Yu HT. A mendelian randomization analysis: the causal association between serum uric acid and atrial fibrillation. Eur J Clin Invest. (2020) 50:e13300. 10.1111/eci.13300 32474920

[74] Zhang CH Huang DS Shen D Zhang LW Ma YJ Wang YM. Association between serum uric acid levels and atrial fibrillation risk. Cell Physiol Biochem. (2016) 38:1589–95. 10.1159/000443099 27082929

[75] Singh JA Yu S. Allopurinol and the risk of atrial fibrillation in the elderly: a study using Medicare data. Ann Rheum Dis. (2017) 76:72–8. 10.1136/annrheumdis-2015-209008 27165177

[76] Zhao J Liu T Korantzopoulos P Letsas KP Zhang E Yang Y. Association between serum uric acid and atrial fibrillation recurrence following catheter ablation: a meta-analysis. Int J Cardiol. (2016) 204:103–5. 10.1016/j.ijcard.2015.11.167 26655551

[77] Papageorgiou N Briasoulis A Lazaros G Imazio M Tousoulis D. Colchicine for prevention and treatment of cardiac diseases: a meta-analysis. Cardiovasc Ther. (2017) 35:10–8. 10.1111/1755-5922.12226 27580061

[78] Imazio M Brucato A Ferrazzi P Pullara A Adler Y Barosi A. Colchicine for prevention of postpericardiotomy syndrome and postoperative atrial fibrillation: the COPPS-2 randomized clinical trial. JAMA. (2014) 312:1016–23. 10.1001/jama.2014.11026 25172965

[79] Liu Z Que S Zhou L Zheng S. Dose-response relationship of serum uric acid with metabolic syndrome and non-alcoholic fatty liver disease incidence: a meta-analysis of prospective studies. Sci Rep. (2015) 5:14325. 10.1038/srep14325 26395162 PMC4585787

[80] Liu T Zhang X Korantzopoulos P Wang S Li G. Uric acid levels and atrial fibrillation in hypertensive patients. Intern Med. (2011) 50:799–803. 10.2169/internalmedicine.50.4587 21498925

[81] Sakabe M Fujiki A Sakamoto T Nakatani Y Mizumaki K Inoue H. Xanthine oxidase inhibition prevents atrial fibrillation in a canine model of atrial pacing-induced left ventricular dysfunction. J Cardiovasc Electrophysiol. (2012) 23:1130–5. 10.1111/j.1540-8167.2012.02356.x 22587612

[82] Choi HK Ford ES. Prevalence of the metabolic syndrome in individuals with hyperuricemia. Am J Med. (2007) 120:442–7. 10.1016/j.amjmed.2006.06.040 17466656

[83] Vural U Aglar AA. What is the role of metabolic syndrome and obesity for postoperative atrial fibrillation after coronary bypass grafting? BMC Cardiovasc Disord. (2019) 19:147. 10.1186/s12872-019-1130-3 31208345 PMC6580605

[84] Polovina M Hindricks G Maggioni A Piepoli M Vardas P Asanin M. Association of metabolic syndrome with non-thromboembolic adverse cardiac outcomes in patients with atrial fibrillation. Eur Heart J. (2018) 39:4030–9. 10.1093/eurheartj/ehy446 30101326

[85] Song P Wang H Xia W Chang X Wang M An L. Prevalence and correlates of hyperuricemia in the middle-aged and older adults in China. Sci Rep. (2018) 8:4314. 10.1038/s41598-018-22570-9 29531237 PMC5847518

[86] Liang WY Liu WW Liu ML Xiang W Feng XR Huang B. Serum uric acid level and left ventricular hypertrophy in elderly male patients with nonvalvular atrial fibrillation. Nutr Metab 3Cardiovasc Dis. (2016) 26:575–80. 10.1016/j.numecd.2016.03.011 27162100

[87] Kivity S Kopel E Maor E Abu-Bachar F Segev S Sidi Y. Association of serum uric acid and cardiovascular disease in healthy adults. Am J Cardiol. (2013) 111:1146–51. 10.1016/j.amjcard.2012.12.034 23352265

[88] Chen Y Xia Y Han X Yang Y Yin X Qiu J. Association between serum uric acid and atrial fibrillation: a cross-sectional community-based study in China. BMJ Open. (2017) 7:e019037. 10.1136/bmjopen-2017-019037 29275349 PMC5770963

[89] Yang Y Zhou W Wang Y Zhou R. Gender-specific association between uric acid level and chronic kidney disease in the elderly health checkup population in China. Ren Fail. (2019) 41:197–203. 10.1080/0886022X.2019.1591994 30973288 PMC6461085

[90] Chugh SS Havmoeller R Narayanan K Singh D Rienstra M Benjamin EJ. Worldwide epidemiology of atrial fibrillation: a Global Burden of Disease 2010 Study. Circulation. (2014) 129:837–47. 10.1161/CIRCULATIONAHA.113.005119 24345399 PMC4151302

[91] Peters SAE Woodward M. Established and novel risk factors for atrial fibrillation in women compared with men. Heart. (2019) 105:226–34. 10.1136/heartjnl-2018-313630 30158135

[92] Kleber ME Delgado G Grammer TB Silbernagel G Huang J Kramer BK. Uric acid and cardiovascular events: a mendelian randomization study. J Am Soc Nephrol. (2015) 26:2831–8. 10.1681/ASN.2014070660 25788527 PMC4625666

